# Neuroprotective Effects of Psychotropic Drugs in Huntington’s Disease

**DOI:** 10.3390/ijms141122558

**Published:** 2013-11-15

**Authors:** Edward C. Lauterbach

**Affiliations:** 1 Department of Psychiatry and Behavioral Sciences, Mercer University School of Medicine, 655 First Street, Macon, GA 31201, USA; E-Mail: eclbgnp@earthlink.net; Tel.: +1-478-745-8531; 2 Department of Internal Medicine, Neurology Section, Mercer University School of Medicine, 707 Pine Street, Macon, GA 31201, USA; 3 Center for Translational Studies In Neurodegenerative Disease, Mercer University, 1400 Coleman Avenue, Macon, GA 31207, USA

**Keywords:** neurodegenerative disease, antipsychotic, mood stabilizer, antidepressant, anxiolytic, hypnotic, gene expression, epigenetics, apoptosis, animal model, Huntington’s disease

## Abstract

Psychotropics (antipsychotics, mood stabilizers, antidepressants, anxiolytics, *etc.*) are commonly prescribed to treat Huntington’s disease (HD). In HD preclinical models, while no psychotropic has convincingly affected huntingtin gene, HD modifying gene, or huntingtin protein expression, psychotropic neuroprotective effects include *upregulated* huntingtin autophagy (lithium), histone acetylation (lithium, valproate, lamotrigine), miR-222 (lithium-plus-valproate), mitochondrial protection (haloperidol, trifluoperazine, imipramine, desipramine, nortriptyline, maprotiline, trazodone, sertraline, venlafaxine, melatonin), neurogenesis (lithium, valproate, fluoxetine, sertraline), and BDNF (lithium, valproate, sertraline) and *downregulated* AP-1 DNA binding (lithium), p53 (lithium), huntingtin aggregation (antipsychotics, lithium), and apoptosis (trifluoperazine, loxapine, lithium, desipramine, nortriptyline, maprotiline, cyproheptadine, melatonin). In HD live mouse models, delayed disease onset (nortriptyline, melatonin), striatal preservation (haloperidol, tetrabenazine, lithium, sertraline), memory preservation (imipramine, trazodone, fluoxetine, sertraline, venlafaxine), motor improvement (tetrabenazine, lithium, valproate, imipramine, nortriptyline, trazodone, sertraline, venlafaxine), and extended survival (lithium, valproate, sertraline, melatonin) have been documented. Upregulated CREB binding protein (CBP; valproate, dextromethorphan) and downregulated histone deacetylase (HDAC; valproate) await demonstration in HD models. Most preclinical findings await replication and their limitations are reviewed. The most promising findings involve replicated striatal neuroprotection and phenotypic disease modification in transgenic mice for tetrabenazine and for sertraline. Clinical data consist of an uncontrolled lithium case series (*n* = 3) suggesting non-progression and a primarily negative double-blind, placebo-controlled clinical trial of lamotrigine.

## 1. Introduction

Huntington’s disease (HD) is caused by mutant huntingtin protein resulting from an expanded polyglutamine cytosine-adenine-guanine (CAG) repeat sequence in the autosomal dominant gene *huntingtin* (*HTT*, 4p16.3) [1]. Mutant huntingtin protein accumulates and produces transcriptional dysregulation, proteasomal, autophageal, mitochondrial, and metabolic dysfunctions, oxidative stress, apoptosis, neuroinflammation, and consequent neurodegeneration, especially in the striatum [2–4].

Besides the movement and dementia syndromes of HD, psychiatric disorders are a fundamental feature of the disease and include personality changes and psychotic, bipolar, depressive, anxiety, impulse control, and other disorders [5–8]. These disorders are commonly treated with a variety of psychotropic agents that include antipsychotics, lithium, anticonvulsants, antidepressants, anxiolytics, and hypnotics during the routine care of patients with HD [5–8].

We have been interested in the effects of commonly used, first-line psychotropic drugs on gene expression [9–11], pathogenic protein metabolism [12–15], proteasomal [12–15] and mitochondrial function [12–15], apoptosis [12–15], inflammation [12,13,15], and other neurodegenerative processes in Alzheimer’s disease [9,12–14], Parkinson’s disease [10–15], and other neurodegenerative diseases [12–14].

Here, the effects of psychotropics on gene expression, huntingtin protein expression, transcriptional dysregulation, and these other neurodegenerative processes that are active in HD are considered. Because it cannot be supposed that the neuroprotective effects of psychotropics in HD models are explained by the psychopharmacodynamic mechanisms of these drugs, the diversity of commonly prescribed psychotropics approved by the United States Food and Drug Administration are investigated here and are listed by class in Table 1.

## 2. *HTT* Gene Expression

Given the relation between HD and the expression of mutant huntingtin protein, downregulation of *HTT* gene expression by psychotropics might be therapeutic. There are additional genes that appear to modify the phenotype of HD, and psychotropic modulation of these genes might also modify disease course, albeit to a much lesser extent than *HTT*. For purposes of this discussion, “*HTT*” refers to the human gene whereas “*htt*” refers to the gene in non-human animals.

### 2.1. *HTT* Gene Expression

Investigation into the reduced expression of mutant huntingtin protein by psychotropics is at a very early stage and initial findings have thus far not shown consistent significant effects. The effects of psychotropics on *HTT* expression have been studied only to a limited degree, and only one study involving fluoxetine has indicated a change in gene expression exceeding the criterion of 20%. In studies of antipsychotics, neither haloperidol nor clozapine produced significant htt expression changes at 4 weeks (by two reporters) or 12 weeks (one reporter) of administration in mouse whole brain gene chip studies (GEO Profiles database data [16], no author cited for either study). The antidepressant fluoxetine was administered to DBA/2J mice at 18 mg/kg/day (about 63 times the human oral dose) for 21 days, upregulating *htt* expression 24% in mouse hippocampus at reporter L23312 by probeset 1425969_a_at, but not at three other reporters (−8% at AW553740 1456667_at, −3% BB411609 1446337_at, +2% BE947966 1435539_at) after 3 weeks treatment [17]. An analysis of probesets revealed comparability and that all probesets had received an annotation grade of A, with the probeset showing change possessing an identity of 97.94 with one cross hybridizing transcript whereas the other three probesets had respective identities of 81.86, 99.85, and 94.9 and had no cross hybridizing transcripts (Affymetrix website [18]). Other gene chip studies including *Htt* in the GEO Profiles database included the antipsychotic olanzapine in rat frontal cortex [19], the mood stabilizer valproate in human ovarian thecal cells at two reporters, and a combination of the monoamine oxidase inhibitor moclobemide with tacrine and creatinine in R6/2 HD transgenic mice at two reporters [16], but absent detection calls precluded analyses of these data.

Thus, at present, the evidence is inconclusive that fluoxetine can meaningfully upregulate *htt* expression at least in mouse hippocampus at very high doses, and haloperidol and clozapine did not appear to affect expression when averaged in whole brain. Other psychotropics, including olanzapine and valproate, require further study for their effects on huntingtin expression. The olanzapine dose (2 mg/kg/day) was about 7 times the usual human therapeutic dose. It should also be noted that whole brain, hippocampus, and frontal cortex do not necessarily represent striatal gene expression effects, and do not necessarily predict effects in HD models or human disease after more chronic drug administration. Furthermore, drug-driven gene expression often varies over time, sometimes with opposite effects at different points in time, and treatment in humans would usually be no less than 6 months in duration and often more than a year for these drugs. Future studies will ideally involve doses and treatment durations relevant to human therapeutics. Gene chip positive findings should be confirmed by real time polymerase chain reaction (RT-PCR) studies. Consequently, the study of psychotropics on *Htt* expression is only in its preliminary stages and, given the frequency at which these drugs are prescribed, further investigation of these and other psychotropics is obviously needed.

In summary, while psychotropics have thus far not been demonstrated to affect *HTT* transcription, this is an early literature and only a few drugs have been studied, and at doses that do not reflect human therapeutic doses or durations of treatment, conditions that can dramatically alter gene transcription findings. Future positive gene chip findings should be confirmed by quantitative RT-PCR analysis.

### 2.2. HD Modifying Influences and Genes, and Their Expression

A number of genes can exert modifying influences on the HD phenotype, particularly its age of onset. It is thought that, besides the *HTT* gene that accounts for about 70% of the variance in age of onset, and other genes account for another 17%, with environmental factors likely explaining the rest [20]. Disease-modifying genetic influences on HD include those affecting HTT CAG repeat number, which is inversely correlated with age of onset, and genes other than *HTT* that may influence HD.

In the *HTT* gene, the HD Δ2642 polymorphism in French populations [21] and the CCG-repeat coding for a polyproline-repeat polymorphism in Eastern Indians [22] have been associated with age of onset. A literature search of gene candidates that might mitigate CAG repeat length within individual HD patients was unrevealing, although a therapeutic effect of downregulating the *HDL2* gene *JPH3* [23] cannot be fully excluded at this point, and intergenerational expansion might potentially be addressed by downregulating *OGG1* [24] or *MSH2*, *MSH3*, and *PMS2* [25], but these are highly speculative considerations.

Genes other than *HTT* may influence HD. Gusella and MacDonald pointed out the many difficulties in substantiating the reported genetic modifiers of HD course, finding small effects and variable replications for genes involved with glutamatergic transmission (*GRIK2*, *GRIN2A*, *GRIN2B*), protein degradation (*UCHL1*), gene transcription (*TCERG1*, *TP53*), stress response/apoptosis (*DFFB*, *MAP3K5*, *MAP2K6*), lipoprotein metabolism (*APOE*), axonal trafficking (*HAP1*), folate metabolism (*MTHFR*), and energy metabolism (*PPARGC1A*), with some showing sex-specific effects (*APOE*, *GRIN2A*, *GRIN2B*, *MAP2K6*), and most associated primarily with age of disease onset [26]. Kalathure *et al.* evaluated the more than 800 genes associated with HD considering their strengths of association and biological functions and determined the most promising therapeutic targets besides *HTT* to be *SUMO1*, *BZW1*, *SF3B1*, *CREB1*, *FZD5*, *DNAJB2*, *CDK5R2*, *ARPC2*, *S100P*, *WDR1*, *ADD1*, *WHSC2*, *HSPA4*, *SKP1*, *ETF1*, *HSPA9*, *UBE2D2*, *HDAC3*, *TCERG1*, *PPARGC1B*, *FRK*, *RPS12*, *MED23*, *MAP3K5*, and *GRM1* [27]. These genes, which include several discussed by Gusella and MacDonald [26], are involved in a variety of cellular mechanisms, some of which include gene transcription (*CREB1*, *WHSC2*, *TCERG1*, *PPARGC1B*, *RPS12*, *MED23*), RNA splicing (*SF3B1*, *MED23*), macromolecular biosynthesis (*ARPC2*, *ADD1*, *HSPA4*, *ETF1*), protein biosynthesis (*ADD1*, *HSPA4*), protein post-translational modification (*SUMO1*, *CREB1*, *ETF1*, *UBE2D2*, *HDAC3*, *FRK*, *MAP3K5*), protein folding (*HSPA9*) and localization (*SUMO1*, *HSPA9*), nucleocytoplasmic transport (*HSPA9*), regulation of protein catalytic activity (*SKP1*, *CDK5R2*, *MAP3K5*), protein phosphorylation (*CREB1*, *CDK5R2*, *FRK*, *MAP3K5*), biopolymer metabolism (*UBE2D2*, *FRK*, *MAP3K5*), protein metabolism (*DNABJ2*, *ARPC2*, *ADD1*, *ETF1*, *HSPA9*, *PPARGC1B*, *FRK*, *MAP3K5*), protein degradation (*SUMO1*, *SKP1*, *UBE2D2*), proteasomal function (*SKP1*, *UBE2D2*), organelle biogenesis (*SUMO1*, *ADD1*, *HSPA4*, *ETF1*, *HDAC3*), membrane organization (*HSPA4*), cell development and differentiation (*FZD5*, *WDR1*, *HSPA9*, *HDAC3*, *MED23*, *MAP3K5*), cell cycle (*CDK5R2*, *SKP1*, *HDAC3*), cell proliferation (*BZW1*, *FRK*), cell migration (*S100P*), synaptic transmission (*S100P*, *GRM1*), cell signal transduction (*CREB1*, *MAP3K5*) including cell surface receptor-linked signal transduction (*FZD5*, *GRM1*), cellular stress responses (*SUMO1*, *DNABJ2*, *HSPA4*, *MAP3K5*), and regulation of apoptosis (*HSPA9*, *HDAC3*, *MED23*, *MAP3K5*) [27]. In particular, *CDK5R2* encodes CDK5 which reduces the cleavage and toxicity of mutant huntingtin, *HDAC3* encodes histone deacetylase 3 (see Section 4.5 below), *TCERG1* encodes the transcriptional coactivator CA150 and has been linked to HD in genome-wide association studies (GWAS), PPARGC1B activates several transcription factors and nuclear receptors that are related to HD dysfunction, and MAP3K5 activates apoptotic kinase cascades in the presence of mutant huntingtin and has also been linked to HD in GWAS [27]. Finally, increased nuclear RE1 Silencing Transcription Factor (REST) in HD represses the transcription of several thousand genes, producing transcriptional dysregulation [28]. Consequently, *REST* downregulation might prove therapeutic in HD.

A review of the published medical literature disclosed no articles in HD models regarding psychotropic effects on HTT CAG repeats, the modifying genes listed above, or *REST*. Although there is no way to know if any of the findings in non-HD models can potentially translate to HD, a few results were found for modifying genes and for *REST* (An analysis of GEO Profiles data for modifying genes other than *HTT* is beyond the scope of this article). In our recent review, antipsychotics were found to downregulate *UCHL1* while the SSRI antidepressant fluoxetine upregulated it [10]. An increase in *MTHFR* promoter activity, mRNA (2.5-fold), and protein (3.7-fold) was observed in HepG2 cells exposed to valproate [29]. Similarly, the same group had earlier found that valproate and lithium each increased *MTHFR* expression in a cellular endoplasmic reticulum stress model [30]. In previous work, we analyzed the murine gene chip data from the GEO Profiles database for *APOE*, finding less than an 8% downregulated transcription after 3 weeks of fluoxetine treatment, less than 2% upregulation after 4 weeks of haloperidol and clozapine treatment, and less than 2% downregulation after 12 weeks of haloperidol and clozapine treatment [10,15]. In differentiated adipocytes, exposure to clozapine increased the mRNA expression of *ADD1/SREBP(1C)* as assessed by RT-PCR [31]. In both adipocytes and hepatocytes, glycogen synthase kinase-3 (GSK-3) inhibited *ADD1/SREBP1c* transcription while GSK-3 inhibitors, which include lithium, increased transcription [32]. Valproate administration increased *SKP1A* gene expression in a steroidogenic *CYP11A1* gene model [33]. In a recent study of psychotropic effects on genes associated with Parkinson’s disease onset and progression, fluoxetine was found to upregulate *Skp1a* expression [11].

In non-HD models, lithium downregulated REST binding and protein levels in a rat fetal alcohol syndrome model [34] whereas valproate upregulated *REST* transcription in medulloblastoma cells [35], but neuroprotectively repressed *REST* in the Niemann-Pick Type C disease NPC1 knockout mouse [36]. Whether these findings will translate to a therapeutic effect of either drug in HD is impossible to predict, especially given the disparate valproate findings in different neuropathological models.

In summary, psychotropics are not known to affect HTT CAG repeat length. Although in non-HD models, antipsychotics can downregulate *UCHL1*, clozapine upregulates *ADD1*, lithium upregulates *MTHFR* and *ADD1*, valproate upregulates *MTHFR* and *SKP1A*, and fluoxetine upregulates *SKP1A* transcription while lithium downregulates REST, although these findings have yet to be demonstrated in HD models, let alone in human HD at conventional therapeutic doses. HD modifying genes continue to be evaluated by ongoing association studies, and the strengths of their modifying effects will vary with continuing incoming data.

## 3. Huntingtin Protein Expression, Aggregation, Autophagy, and Proteosomal Function

More important than *HTT* gene expression is the huntingtin protein concentration to which cells are exposed, determined by protein expression, aggregation, and autophagic and proteasomal clearance from the cell. Psychotropic modulation of these processes would therefore be of obvious importance in an attempt to limit HD pathobiology. While there have been no reports of psychotropics directly influencing huntingtin protein expression, several studies have considered aggregation and autophagic processing.

Most antipsychotics have pronounced dopamine D2 receptor antagonist properties. Charvin and colleagues found that dopamine increased mutant huntingtin aggregates in all cellular compartments in primary striatal neuronal cultures, an effect that was reversed by 1 μM D2 receptor antagonist [37], about 20 times human therapeutic concentrations, and this effect was also observed in an animal model (see Section 8).

Carmichael *et al.* found the mood stabilizer lithium at 2.5–5 mM for 3 days (2–5 times human therapeutic plasma levels) to reduce mutant huntingtin nuclear inclusions and apoptotic nuclear fragmentation in COS-7 African green monkey kidney cells and SKNSH human neuroblastoma cell lines transfected with mutant *HTT* exon 1 fragment possessing 74 CAG repeats [38]. Sarkar and colleagues demonstrated 10 mM lithium to enhance the autophagy of mutant huntingtin in COS-7 [39], SKNSH [39], PC12 [39], and mouse embryonic fibroblast [40] cells transfected with mutant *HTT* exon 1 with 74 CAG repeats [39–41]. Lithium can actually have mixed effects on autophagy, enhancing it by downregulating glycogen synthase kinase-3β (GSK-3β) [38], inositol species [39,41,42], or cytoplasmic p53 [43], or, at higher doses, reducing autophagy by activating the mammalian target of rapamycin (mTOR) through GSK-3β inhibition [40]. Striatal GSK-3B may be upregulated in HD since serine 9 phosphorylation of GSK-3β negatively regulates GSK-3β, and it was shown that this phospho-GSK-3β was reduced in both the striatum and cerebral cortex at the onset of behavioral manifestations in both N171-82Q and YAC128 HD transgenic mice [44]. Although not in HD models but rather in Parkinson models, lithium, valproate, and carbamazepine each increased autophagy markers (lysosome number, autophagic vacuolar organelle number, and microtubule-associated protein 1 light chain 3-II (LC3-II) expression) and reduced apoptosis in rotenone-exposed SH-SY5Y cells [45], and lithium-plus-valproate increased LC3, protected nigrostriatal dopamine neurons, and improved motor outcomes that were attributed to autophagic and lysosomal pathway activation in the MPTP mouse model [46].

Peng and colleagues administered clinically relevant doses of the antidepressant sertraline (5–10 mg/kg/day, about 3.5 times the human therapeutic dose) for 10 weeks to R6/2 HD transgenic mice but did not find reductions in intranuclear aggregated huntingtin protein in striatal, hippocampal, or cortical neurons [47]. Similarly, a trial of 5–20 mg/kg/day sertraline (about 6 times human therapeutic doses) for 8 weeks in the N171-82Q HD transgenic mouse model revealed no improvement in intranuclear huntingtin inclusions even though striatal atrophy was reduced and neurogenesis and survival were increased relative to control vehicle-treated N171-82Q mice [48].

Thus far, D2 antagonists, lithium, and sertraline have been studied, drugs which are frequently employed in HD for the psychotic, bipolar, and depressive features of the disease. Overall, the evidence in HD models suggests that, while no psychotropics have been demonstrated to directly affect huntingtin expression or proteasomal degradation, haloperidol and lithium may have the potential to stimulate autophagy and reduce huntingtin aggregates. Haloperidol reduced cellular aggregates in a cell culture and a transfected rat model at levels 20 and 200 times human therapeutic doses, respectively. Haloperidol effects on huntingtin aggregation should now be replicated at clinically relevant doses and studied further in additional HD models. Lithium reduced nuclear huntingtin aggregates in 2 cell models and stimulated autophagy of huntingtin in at least 4 different cell models at doses 2–10 times human therapeutic levels. Lithium inhibits presumably overly active GSK-3β that has at least been observed in HD transgenic mouse models. It will be of interest to replicate the findings for lithium at lower human therapeutic concentrations in human striatal cells expressing mutant huntingtin. The SSRI antidepressant sertraline failed to influence aggregates in either of two different HD transgenic mouse models. Sertraline’s lack of effect on huntingtin intranuclear aggregates in the R6/2 mouse, however, may not translate to human HD because R6/2 truncated human mutant huntingtin in the mouse produces a different subcellular localization than the non-truncated protein in actual HD [49].

In summary, besides haloperidol, lithium, and sertraline, other psychotropics await study. The effects of haloperidol and lithium on huntingtin aggregation and autophagy demonstrated in cell culture should now be studied in transgenic models at doses that produce blood levels corresponding to human therapeutic concentrations.

## 4. Epigenetics

Beyond downregulating *HTT* gene transcription or huntingtin concentrations, it may be possible to reverse the effects of mutant huntingtin. It would therefore be of therapeutic interest to determine whether psychotropics can epigenetically repair the transcriptional dysregulation wrought by huntingtin protein. Putative epigenetic targets are listed in Table 2 and targets for which psychotropic treatment data are available are discussed below, including AP-1, histone acetylation, CBP, p53, HDAC inhibition, miRNA, and eRNA.

Findings in human HD and HD transgenic models were reviewed to generate putative epigenetic targets that might be addressed by psychotropics, provided in Table 2 (Epigenetic Targets In Huntington’s Disease). It should be understood that some of these targets may represent epiphenomena unrelated to HD pathogenesis. Overall, there is evidence that mutant huntingtin impairs the transcription of normal neuronal genes while de-repressing dedifferentiating genes [4]. Consistent with data from HD models, most targets listed in Table 2 are generally associated with increased gene transcription. DNA methylation, however, is a putative target that tends to repress gene transcription, and some explanation is therefore needed. A single first-of-its-kind cell culture study found net DNA demethylation (61,940 cytosines demethylated and 33,974 cytosines hypermethylated of 97,006 cytosines assessed) in striatal cells expressing 111-CAG repeat mutant HTT in comparison to striatal cells expressing non-pathogenic 7-repeat HTT [50]. DNA demethylation was present primarily (84%) in low cytosine-followed-by-guanine sequence regions (low CpG regions) in intergenic areas that are rich in gene promoters and enhancers [50], in contrast to the more usual high CpG region intragenic methylation. Thus, while it would seem desirable to demethylate genes in CpG-rich areas to improve transcription based on the bulk of epigenetic targets derived from other HD model data, the available experimental evidence indicates that demethylation of intergenic CpG-poor regions is already prominent in this model of HD. Based on this evidence, an epigenetic target would therefore be upregulation of DNA methylation particularly in CpG-poor intergenic areas. Perhaps such methylation may repress dedifferentiating and pro-apoptotic genes and de-repress pro-survival genes since, in contrast to gene repression in CpG-rich areas, methylation in CpG-poor areas is associated with both decreased and increased transcription [50].

Several caveats apply in interpreting epigenetic target findings. These targets are putative and, as mentioned above, some of the targets may represent epiphenomena unrelated to HD pathogenesis. A present difficulty is that it is not yet clear which psychotropics that upregulate DNA methylation or other transcriptional repression do so in CpG-poor areas, and this must be clarified before epigenetic neuroprotective predictions can be considered valid. In the event that DNA-methylating psychotropics act primarily in CpG-rich areas, such drugs might actually promote HD progression. Another problem is that drugs that act one way in normal neurons may act in opposite fashion under pathological conditions. For example, lithium and valproate usually upregulate AP-1 DNA binding activity but they neuroprotectively downregulate AP-1 DNA binding activity under conditions of toxic glutamate and kainate exposure, respectively, as seen below (Section 4.1). Still another concern is that there are epigenetic differences between human HD and HD transgenic mouse models. For instance, the histone H3 is hyperphosphorylated in human HD [105] yet hypophosphorylated in the R6/2 HD transgenic mouse [66]. Furthermore, epigenetic findings may differ dramatically between the early and late stages of HD, or according to the duration of treatment. There are also examples of discrepancies between therapeutic predictions derived from HD models and experimental observations in HD models, for example p53 (see Section 4.4) and certain microRNA (see Section 4.6.2). Thus, it is necessarily premature to draw anything but tentative conclusions about the epigenetic therapeutic potential of psychotropics until candidate drugs can be systematically studied in human HD striatal cell lines, or at least in multiple HD transgenic animal models. Until then, the present state of the scant HD-related model evidence for the array of HD model-based epigenetic targets is provided below.

### 4.1. AP-1 DNA Binding Activity Downregulation

As explained above, upregulated DNA methylation appears to constitute a therapeutic goal in HD, and reduced AP-1 binding would be consistent with this goal. FRA-2 and JUND are components of the activator protein 1 (AP-1) complex and their increased DNA binding is associated with decreased DNA methylation in striatal cells expressing mutant *HTT* [50]. This suggests that a reduction in AP-1 DNA binding activity might lead to increases in DNA methylation. It will be important to replicate the findings of this study both within the striatal cell model used and in other HD models, and using mutant Htt of different repeat lengths. Moreover, the direction of any causal relationship between AP-1 binding activity and DNA methylation needs to be established.

In an HD-related model of glutamate NMDA-mediated excitotoxicity, lithium 0.5–2 mM (human therapeutic levels are 0.5–1.5 mM/L) for 7 days neuroprotectively downregulated AP-1 DNA binding, antagonizing glutamate-induced activation (phosphorylation) of JNK, p38 kinase, and subsequent AP-1 binding and apoptosis in rat cerebellar granule cells [106]. Thus, lithium neuroprotectively downregulated AP-1 binding in this glutamate cell model and this accords with lithium’s neuroprotective downregulation of AP-1 binding activity in non-HD models including ethanol fetal toxicity [107], ischemia [108], and carbachol stimulation [109], in stark contrast to lithium’s upregulating effect on AP-1 binding in non-neuropathological models [110–117]. In neuropathological non-HD models, other drugs neuroprotectively downregulating AP-1 DNA binding activity include valproate in the kainic acid seizure model [118], carbamazepine in the carbachol stimulation model [109], and diazepam [118] and dextromethorphan [119–121] in the kainic acid seizure model while none pro-apoptically upregulated AP-1 DNA binding.

This initial lithium study [106] at human therapeutic levels requires both replication within this glutamate model and confirmation in other HD models. While kainic acid exposure has been used as an HD model, it is difficult to know if the findings for valproate, diazepam, and dextromethorphan might also be obtained in formal HD models since the kainate data are confounded by the additional presence of seizures. Of relevance to HD, dextromethorphan has been noted to downregulate the transcription of many apoptotic genes that are induced by glutamatergic NMDA receptor activation [122], although it is unknown if this drug can remediate the transcriptional dysregulation of HD. Valproate, carbamazepine, diazepam, and dextromethorphan should be tested in HD models. In non-HD models, trifluoperazine upregulated AP-1 DNA binding while haloperidol, clozapine, lithium, valproate, imipramine, and amitriptyline downregulated AP-1, but these findings do not necessarily predict outcomes in HD models, as is seen particularly in the case of lithium, and these drugs should be investigated in HD paradigms.

In summary, a single finding of putatively neuroprotective neuronal AP-1 DNA binding downregulation occurring at human therapeutic lithium concentrations in a cell culture exposed to glutamate should now be replicated and studied in models involving mutant huntingtin. Beyond this single lithium study, other psychotropics await study in HD models.

### 4.2. Histone H3 and H4 Acetylation Upregulation

Numerous studies have documented H3 and H4 hypoacetylation in HD animal models [57–60] and in human HD [55,61]. Although striatal histone acetylation was elevated in patients with HD in the study of Anderson *et al.* 2008, the authors suggested that this could represent an artifact of neuronal loss, astrocytosis, and microglial activation [123]. Consequently, the weight of the evidence suggests that upregulating H3 and H4 acetylation may improve the transcriptional dysfunction present in HD.

In both N171-82Q and YAC128 HD transgenic mice, lithium and valproate each increased histone H3 acetylation at lysines 9 (H3K9) and 14 (H3K14) in both the striatum and cortex, with combined lithium and valproate treatment leading to more robust increases in N171-82Q mice whereas this was evident only in YAC128 striatum but not cortex [44]. Animals were fed dietary lithium 3 g/kg/d and valproate 25 g/kg/day throughout their life beginning at week 7, producing human therapeutic serum levels of 0.8–1.2 mM/L and 73–94 μg/mL, respectively, in both mouse models. Lamotrigine was found to increase histone acetylation in an HD-related glutamate excitotoxicity model. In rat cerebellar granule cells exposed to glutamate excitotoxicity, pretreatment with lamotrigine 0.1 mM or valproate 1 mM (about twice human therapeutic concentrations for these drugs) each neuroprotectively upregulated histone H3 and H4 acetylation as well as anti-apoptotic Bcl-2 levels and inhibited glutamate excitotoxicity through upregulated histone acetylation and activity of the Bcl-2 promoter [124]. A sub-effective lamotrigine dose given with either lithium or valproate provided synergistic neuroprotection [124].

In non-HD models, fluoxetine protectively upregulated H3 acetylation in methyl-mercury [125] and social defeat [126] paradigms.

Although the lamotrigine study needs replication in mutant huntingtin cell cultures and other HD models, this drug has already been studied in an HD clinical trial, with negative findings (see Section 9). Fluoxetine should be studied in an HD model and positive results should then be confirmed across other HD models, especially human cell lines and transgenic animals. The lithium and valproate findings should be explored in additional HD models. In non-HD models, olanzapine, clozapine, lithium, valproate, lamotrigine, amitriptyline, clomipramine, escitalopram, and duloxetine have upregulated H3 acetylation while fluoxetine has downregulated it, and amphetamine, carbamazepine, valproate, topiramate, and fluoxetine have upregulated H4 acetylation, but again, such findings do not necessarily predict outcomes in HD.

In summary, lithium and valproate each neuroprotectively increased striatal and cortical histone H3K9 and H3K14 acetylation at human therapeutic concentrations in two HD transgenic mouse models while lamotrigine and valproate each increased neuronal H3 and H4 acetylation in a single glutamate cell culture model, although lamotrigine failed to meet endpoints for HD disease modification in a human clinical trial. Lithium and valproate findings should be replicated within and across transgenic mouse models and might also be explored in human HD peripheral blood.

### 4.3. CBP Upregulation

CREB-binding protein CBP (KAT3A) is a histone acetyltransferase that is sequestered by mutant huntingtin [57,58,64], suggesting that transcriptional dysregulation in HD might be improved by upregulating CBP. Although not employed in an excitotoxic model, dextromethorphan is a non-competitive NMDA receptor antagonist and was found to upregulate CBP expression and downregulate the expression of many glutamate-induced apoptotic genes [122]. Valproate neuroprotectively upregulated CBP in an ALS SOD1 transgenic mouse model [127] whereas citalopram prevented stress-induced CBP mRNA upregulation in rats [128]. No other studies of psychotropics were apparent. Since these findings were in non-HD models, they remain to be examined in human cell lines and transgenic animal models of HD.

### 4.4. p53 Upregulation

Mutant huntingtin binds the transcription factor p53 to sequester it in polyglutamine aggregates and downregulates its activity to repress transcription [58], suggesting that increasing p53 transcription and concentrations may be therapeutic in HD. p53 may act as a transcription factor for miRNA, and reduced p53 availability in HD contributes to miRNA downregulation in HD [91]. In light of this, although p53 has been associated with apoptosis, nuclear p53 upregulation (as opposed to cytoplasmic p53, which inhibits autophagy, as discussed in Section 3) may be a worthwhile epigenetic target specifically in HD.

In striking contrast, lithium downregulated p53 while affording neuroprotection in an HD-relevant glutamate NMDA-mediated excitoxic model. At least 5 days treatment with lithium, but not more acute treatment, induced dose-dependent decreases in mRNA and protein levels of p53 and apoptotic Bax while increasing cytoprotective Bcl-2 mRNA and protein levels in cultured rat cerebellar granule cells [129]. Seven-day pre-treatment with lithium 0.5–3 mM (human therapeutic serum levels are 0.5–1.5 mM/L) for 7 days prevented glutamate-induced increases in apoptogenic p53, Bax, and cytochrome c release, activations (phosphorylations) of JNK, p38, and p53, and reductions in Bcl-2, and lithium maintained elevated Bcl-2 levels despite glutamate excitotoxic exposure in these cells [106,129]. However, despite neuroprotecting SKNSH cells transfected with mutant HTT, lithium at 2.5–5 mM for 3 days affected neither p53 nor Bcl-2 levels in another study [38]. In an Alzheimer model, lithium again neuroprotectively downregulated p53 in response to beta-amyloid 25–35 in glia [130]. No psychotropics upregulated p53 or its phosphorylation in neurodegenerative models. In non-HD models, haloperidol, valproate, and fluoxetine upregulated p53, and lithium and valproate upregulated p53 phosphorylation (activation), while no psychotropics downregulated p53 or its activation, but these findings are not necessarily predictive of effects in HD.

Lithium should be studied in other HD models given its inconsistent effects on p53 in the two HD models above. It is difficult to assess the relative contribution of p53 to cellular survival since Bcl-2 may have been the driving sustaining force, overshadowing p53-related effects. In non-HD models, lithium has demonstrated mixed effects, including upregulating p53 in bovine aortic endothelial cells [131] and degrading p53 in human HCT116 colon cancer cells [132] and attenuating p53 levels in human SH-SY5Y cells [133]. Even if p53 and p53 activation upregulation are found to promote neurodegeneration as a stand-alone intervention in the presence of mutant huntingtin, they may still constitute therapeutically desirable targets in a multi-drug regimen that addresses other epigenetic disturbances in HD. In rat R6 fibroblasts, lithium increased extracellular signal-regulated kinase (ERK) phosphorylation to upregulate p53 levels but also increased p38 phosphorylation to downregulate p53 levels [134]. Thus, lithium’s effect on p53 may depend on its relative effects on ERK and p38 activation. The neuroprotective *vs.* pro-apoptotic outcome of p53 upregulation may depend on a nuclear (*vs*. cytoplasmic) disposition, the relative balance of ERK and p38 activation, other factors, or the genes that will be activated in the context of *Htt* transcriptional dysregulation. Consequently, it remains possible that p53 upregulation is still an overall worthwhile goal, but further study in HD models is necessary to unravel its importance independent of its relationship to Bax, Bcl-2, and these other factors, especially in HD models. Haloperidol, valproate, fluoxetine, and other psychotropics should now be investigated in HD models.

In summary, lithium effects on p53 in HD models are inconclusive. In contrast to anticipated neuroprotective p53 upregulation, lithium downregulated p53 at human therapeutic concentrations in a glutamate-exposed neuronal model and failed to affect p53 at twice this dose in a mutant huntingtin-transfected neuroblastoma model although lithium was neuroprotective in both experiments. Thus, these findings should be further replicated, and additional study of lithium’s effect on nuclear p53 and p53 independent of Bcl-2 (perhaps by using lentiviral-mediated Bcl-2 silencing RNA [124]) should be carried out in neuronal cultures exposed to mutant huntingtin before pursuing p53 in other HD models. Additionally, other psychotropics, beginning with haloperidol, valproate, and fluoxetine, should be studied in the same manner.

### 4.5. HDAC Downregulation

Histone deacetylase (HDAC) inhibitors (HDACIs) have proven effective in producing neuroprotection in HD transgenic *Drosophila* [57] and N171-82Q mouse [59] models, reducing the deacetylation of already hypoacetylated histones in human HD [76]. Additionally, the HDACI phenylbutyrate also decreased histone methylation in N171-82Q HD transgenic mice [59]. Valproate is renowned as a HDACI. In the N171-82Q mouse model, combined treatment with dietary lithium and valproate at doses corresponding to human therapeutic levels (see Section 4.2) increased histone H3 acetylation and improved motor, behavioral, and survival outcomes [44]. H3 acetylation in both cortex and striatum was increased most with combined treatment, followed by valproate and then lithium over 8 weeks of treatment [44]. In this study, valproate in isolation also extended mouse survival time while lithium in isolation improved motor performance [44]. In cultured cerebellar granule cells, 0.1 mM lamotrigine (twice human therapeutic levels) has also been associated with a 20% reduction in HDAC activity [124]. Studies of lithium and valproate need to now be replicated within this model and extended across other HD transgenic models. HDAC enzymes 1–6 and 11 have been implicated in HD, and several psychotropics have been found to downregulate these enzymes or their function in non-HD models (Table 3), but whether these drugs can therapeutically inhibit these HDAC enzymes in HD models remains a subject for study.

In summary, neuroprotective inferred downregulation of HDAC activity by valproate and, possibly, lithium at human therapeutic levels should now be directly demonstrated and then replicated within the N171-82Q mouse and across other HD transgenic models. Initial studies in either neuronal cultures exposed to mutant huntingtin or HD transgenic models should be considered for lamotrigine, carbamazepine, and amitriptyline, although lamotrigine and carbamazepine can also upregulate HDACs 2, 3, and 5 and both drugs are older agents that are infrequently used in psychiatric practice.

### 4.6. MicroRNAs

#### 4.6.1. MiR-222 Upregulation

MicroRNAs regulate the transcription of myriad genes and are deranged in HD, likely contributing substantially to the transcriptional dysregulation and neuronal dedifferentiation observed in this disease. Of all the miRNA targets in HD considered in Table 2, only miR-222 has thus far been demonstrated to be affected by psychotropics in HD or HD models. MiR-222 was found to be downregulated among 90 miRNAs studied in striatal STHdhQ111/HdhQ111 mouse cells expressing the full-length *Htt* gene with 111 glutamate repeats [95] and also in both 10 week-old R6/2 and 12 month-old YAC128 HD transgenic mice [96]. The only study addressing psychotropics and miR-222 employed combined pre-treatment with 3 mM lithium-plus-0.8 mM valproate (about 3 and 1.5 times human therapeutic lithium and valproate levels, respectively) for 6 days in glutamate-exposed rat cerebellar granule cells, resulting in neuroprotection from excitotoxicity and miR-222 upregulation that was confirmed by RT-PCR [146]. These two drugs now need to be studied individually to determine effects upon miR-222 in this and other HD models. The miRNA profiles of other psychotropics in HD models await investigation.

#### 4.6.2. Other MicroRNA Therapeutic Approaches

The miRNAs evaluated above derive from aberrant levels found in HD and HD models, and some may be merely epiphenomenal. More recently, the modulation of certain miRNAs is now being considered for treating HD disease progression. MicroRNA-34b is significantly elevated in presymptomatic HD plasma, increased in response to mutant *HTT* exon 1, and its blockade increases mutant *HTT* exon 1 toxicity by antagonizing pro-survival activity in pluripotent NT2 cells [93]. In the STHdhQ111/HdhQ111 striatal cell model of HD, exogenous expression of miR-214, miR-150, miR-146a, and miR-125b reduced mutant huntingtin expression and aggregation whereas mutations to these miRNAs prevented, and loss of function reversed, their effect [147]. MicroRNA-22 targets and regulates *HDAC4*, *REST corepressor1*, and *G-protein signaling 2* (*Rgs2*) and reduces caspase activation related to its other targets of proapoptotic *MAPK14/p38* and *Tp53inp1*, and its overexpression inhibited neurodegeneration in primary striatal and cortical cultures exposed to the mutant Htt171-82Q fragment [97]. *In vitro*, in HD transgenic mice, and in induced pluripotent stem cells derived from a patient with HD, miR-196a reduced mutant huntingtin and its aggregation, reducing neuropathological progression and phenotypic behavior [102]. *HTT* gene expression is regulated by miR-137, miR-148a, and miR-214, with *HTT* mRNA concentrations reduced by 40%–50% in HEK293T cells after transfection with each of these microRNAs [101]. These functional data support some (miR-22, miR-125b, miR-146a, miR-150) and contradict other (miR-34b, miR-148a, and miR-214) Table 2 miRNA targets.

The transcription repressor element-1 (RE1) silencing transcription factor (REST, also known as neuron-restrictive silencing factor (NRSF)), is unable to be bound by mutant huntingtin and this leads to increased nuclear REST repression of neuronal specific genes that results in neuronal dedifferentiation [87] and downregulated expression of a number of miRNAs [90,91,100]. Not only does REST downregulate the expression of miR-9 and miR-9*, but miR-9 targets REST and miR-9* targets CoREST, thereby inhibiting the REST silencing complex [90].

Based on these findings, upregulation of miR-9, miR-9*, miR-22, miR-34b, miR-125b, miR-137, miR-146a, miR148a, miR-150, miR-196a, and miR-214 may have therapeutic potential against mutant HTT, REST, HDAC4, apoptosis, and other pathobiological factors in HD. There do not appear to be any studies of psychotropic effects on any of these microRNAs in HD models or other models. Several reports regarding miR-34a and miR-146a involve the use of pilocarpine and lithium together to induce seizures, preventing any inferences about lithium alone. Although in non-HD models, lithium downregulated REST binding and protein levels in a rat fetal alcohol syndrome model [34] whereas valproate upregulated REST transcription in medulloblastoma cells [35] but neuroprotectively repressed REST in the Niemann-Pick Type C disease NPC1 knockout mouse [36]. Whether these findings will translate to a therapeutic effect of either drug in HD is impossible to predict, especially given contradictory valproate findings in different neuropathological models.

In summary, there are no data regarding individual psychotropic drug effects on miRNA or REST in HD models at present. Putatively neuroprotective miR-222 upregulation with combined lithium-valproate treatment should be replicated within the glutamate neuronal model, and each drug should be studied individually in this same model and then in neurons exposed to mutant huntingtin. The effects of psychotropics on the other miRNAs listed in Table 2, particularly miR-9, miR-9*, miR-22, miR-34b, miR-125b, miR-137, miR-146a, miR148a, miR-150, miR-196a, and miR-214, as well as on REST, deserve study in HD models.

### 4.7. Enhancer RNA (eRNA)

Enhancer RNAs (eRNAs) may have the potential to treat transcriptional repression in HD. eRNAs have recently been discovered, enhance transcription of multiple genes, and some are produced by p53 binding in a single intergenic non-coding region, resulting in the enhanced transcription of multiple genes [148]. At present, however, there are no studies of eRNA that regard the use of psychotropics in any model, HD or otherwise, and investigative opportunities remain wide open.

## 5. Mitochondrial Respiration, Permeability Transition Pore, and Cytochrome C Release

Mitochondria are critical to cellular energy production and cell survival. Since mitochondria are impaired in HD, and mitochondrial demise generally leads to apoptotic cell death, mitochondrial protectant properties of psychotropics are of therapeutic interest in HD.

In primary striatal cultures exposed to the *N*-terminal fragment of mutant huntingtin, the D2 antagonist antipsychotic haloperidol (concentration unclear) blocked dopamine-induced fatal downregulation of mitochondrial complex II protein that is associated with neuronal death [149]. The antipsychotic trifluoperazine and antidepressants nortriptyline, desipramine, and maprotiline each inhibited glutamate-induced mitochondrial permeability transition pore development at 2 μM concentrations (about 13 and 4 times human trifluoperazine and antidepressant therapeutic concentrations, respectively), demonstrated indirectly from caspase findings in YAC128 transgenic mouse medium spiny neurons [150]. Nortriptyline also inhibited cytochrome c release in this same HD mouse model study [150]. Sertraline 10 mg/kg/day *p.o.* (about 5 times human therapeutic doses) for 14 days was found to reverse striatal nitrite concentrations, lipid peroxidation, and mitochondrial enzyme dysfunctions in the mitochondrial toxin 3-nitropropionic acid rat model of HD [151]. Similarly, two different studies demonstrated that the antidepressants sertraline and venlafaxine at 20 mg/kg/day each improved striatal glutathione redox status, whereas imipramine 20 mg/kg/day, trazodone 20 mg/kg/day, sertraline 10 or 20 mg/kg/day, and venlafaxine 10 or 20 mg/kg/day each improved mitochondrial respiratory complex activity after 2 weeks in 3-nitropropionate rats [152,153], doses that are 2–5 times human therapeutic doses. The hypnotic melatonin 5 μM for 18 h (at least 100 times human therapeutic levels) corrected mutant huntingtin-induced mitochondrial melatonin-1 receptor loss and the subsequent release of cytochrome c in ST14A striatal cell cultures [154].

In summary, there is preliminary evidence of mitochondrial protective effects for antipsychotics (haloperidol, trifluoperazine), antidepressants (imipramine, nortriptyline, desipramine, maprotiline, trazodone, sertraline, venlafaxine), and the hypnotic melatonin in a number of different HD models that include several huntingtin striatal cultures, YAC128 transgenic mice, and the 3-nitropropionate rat. There is replicated evidence of high-dose sertraline and venlafaxine attenuation of free radicals and improved mitochondrial respiratory status with high doses of sertraline, venlafaxine, imipramine, and trazodone in the nitroproprionate rat, although it remains to be seen whether these results extend beyond this model. The remaining single studies of psychotropic neuroprotective mitochondrial effects across a diversity of mitochondrial indices in a variety of different HD models should now each be replicated within the original model and then extended to other HD models at conventional human therapeutic doses.

## 6. Apoptosis

Besides the other pathobiological effects of mutant huntingtin, reversal of the apoptotic cell death cascade may independently offer the potential to slow the progression of HD, and psychotropic antiapoptotic effects are therefore of interest. Loxapine (1–10 μM) and cyproheptadine (1–10 μM) each blocked caspase-3 activation and rescued mouse striatal cells expressing mutant *Htt* (exon 1 with 111 repeats) from cell death in the Hdh (Q111/Q111) mouse striatal cell model, thought to be mediated by transient phosphorylated activation of ERK that was subsequently associated with reduced neurodegeneration and improved motor performance in R6/2 HD transgenic mice treated with the related drug pizotifen [155]. The antipsychotic trifluoperazine (2 μM) inhibited glutamate-induced apoptosis in YAC128 mouse medium spiny neurons [150] at doses about 13 times human therapeutic concentrations. Lithium 2.5–5 mM (2–5 times human therapeutic concentrations) for 3 days reduced apoptotic nuclear fragmentation in both COS-7 African green monkey kidney cells and SKNSH human neuroblastoma cell lines transfected with mutant *Htt* (exon 1 fragment with 74 CAG repeats), thought to be mediated by GSK-3β inhibition since similar effects were observed with the GSK-3β inhibitor SB216763 and *GSK-3β* knockdown [38]. Lithium 0.5–3 mEq/kg s.c. given 24 h before and 1 h after toxic exposure (producing lithium levels comparable to human therapeutic concentrations) inhibited caspase-3 mediated apoptosis in rat striatum infused with quinolinic acid in the excitotoxic quinolinate rat model of HD [156]. The antidepressants nortriptyline, desipramine, and maprotiline each inhibited glutamate-induced apoptosis at 2 μM concentrations (about 4 times human therapeutic concentrations) in YAC128 mouse medium spiny neurons [150]. Melatonin 5 μM (at least 100 times human therapeutic levels) for 18 h inhibited mutant huntingtin-induced caspase activation in ST14A striatal cell cultures [154].

In summary, there are a number of initial studies of antipsychotics (trifluoperazine, loxapine), lithium, antidepressants (desipramine, nortriptyline, maprotiline), and hypnotics (cyproheptadine, melatonin) indicating neuroprotective effects against apoptotic processes at clinically relevant concentrations across several HD striatal cell culture models (YAC128 transgenic mouse striatal medium spiny neurons, striatal cells exposed to mutant huntingtin or quinolinate). Each of these promising findings should now be replicated within its original model and then extended in other HD models, albeit at conventional human therapeutic doses.

## 7. Neurogenesis

Brain derived neurotrophic factor (BDNF) is essential for the survival and neurogenesis of striatal neurons, the principal pathologic substrate of HD, and BDNF is depleted in HD. The effects of psychotropics on BDNF and related neurogenesis therefore warrant consideration.

Lithium at clinically relevant doses (see Section 6) stimulated striatal neuronal and astroglial progenitor proliferation in the HD striatal quinolinate rat model [156]. Ultra-low dose lithium (lifelong 20–40 μg/kg/day per rectal mucosal absorption beginning at 2 months) reversed BDNF deficits in the YAC128 HD transgenic mouse [157]. This dose produces lithium levels 500–1000 times lower than human therapeutic concentrations [157]. In both N171-82Q and YAC128 HD transgenic mice, lithium and valproate each increased BDNF mRNA and BDNF protein in both the striatum and cortex at doses corresponding to human therapeutic levels (see Section 4.2), although the cell-surface BDNF receptor TrkB was increased only by valproate in N171-82Q striatum and cortex and only by combined lithium and valproate treatment in YAC128 striatum [44]. Previous work by this group indicated that GSK-3β inhibition by 1 mM lithium and HDAC inhibition by 0.5 mM valproate (*i.e.*, human therapeutic lithium and valproate concentrations) activate BDNF promoter IV in cortical neurons, potentially explaining these findings [158]. In the R6/1 HD transgenic mouse expressing mutant *Htt* exon 1 with 115 CAG repeats, fluoxetine 20 mg/kg i.p. (at least 100 times human oral therapeutic doses) beginning from 10 weeks till 20 weeks of age rescued neurogenesis and reversed volume loss in the hippocampal dentate gyrus, although it did not produce a net increase in cell proliferation [159]. The antidepressant sertraline given for 10 weeks at clinically-relevant albeit high doses (see Section 3) enhanced striatal neurogenesis and increased BDNF brain levels in R6/2 mice [47]. Sertraline similarly dosed (see Section 3) also increased striatal neurogenesis and BDNF levels in the N171-82Q HD transgenic mouse model [48]. The neurogenesis findings for lithium and fluoxetine deserve replication, and the effects of lithium and sertraline on BDNF and sertraline neurogenesis should now be studied across additional HD models. An analysis of BDNF mRNA and protein levels in the blood of patients with HD and presymptomatic HD unfortunately revealed no significant differences from human healthy controls [160], indicating that peripheral assessments of BDNF in patients with HD will not be suitable for future investigations.

In summary, at clinical doses, lithium, valproate, and sertaline have each increased striatal BDNF levels (and sertraline increased striatal neurogenesis) in two different HD transgenic mouse models while single studies indicate lithium stimulated striatal neurogenesis in the quinolinate rat and that very high dose fluoxetine stimulated hippocampal neurogenesis and preserved hippocampal volume in the R6/1 mouse. Each finding awaits within-model replication, whereupon lithium, valproate, and sertraline should be studied in additional transgenic mice and considered for extension into human HD investigations.

## 8. Animal Models

Although cell and tissue model studies can yield promising findings, such findings do not always translate to disease modifying effects in animal models and humans. The ability of psychotropics to modify the HD phenotype in animal models is therefore important to consider.

Chronic treatment with haloperidol decanoate (30 mg and 50 mg i.m. every 3 weeks, about half the actual number of milligrams used in treating schizophrenia in humans) protected striatal neurons from mutant huntingtin-related dysfunction and extranuclear huntingtin aggregation at both 2 and 8 weeks of treatment in rats transfected with lentivirus expressing mutant HTT171-82Q [161]. The vesicular monoamine transporter-2 inhibitor tetrabenazine 0.25 mg/week (about 25% of the human therapeutic dose) for 8 months beginning at 2 months of age decreased medium spiny neuron loss and improved motor performance in the YAC128 HD mouse model [162]. In another YAC128 mouse study, tetrabenazine 0.375 mg/week begun at either 2 months or 6 months of age reduced striatal cell loss and motor deficits in both groups [163].

Chronic lithium administration (rearing in the presence of 4.2 mM lithium) reduced optic photoreceptor neuronal degeneration in the gmr-httQ120 HD *Drosophila* model expressing the first 171 residues of mutant Htt with 120 repeats, and combining lithium with the mTOR inhibitor rapamycin produced still greater neuroprotection than either drug alone [40]. Lithium (0.3% in chow for 3 weeks, producing blood levels of 0.9 mEq/L that are therapeutic in humans) reduced striatal neuronal degeneration and lesion size, attenuated weight loss, and prolonged survival in the 3-nitropropionic acid rat model of HD [164]. Lithium inhibited striatal neuronal loss in the HD quinolinate rat model at clinically relevant doses (see Section 6) of 0.5–3 mM/kg [156], and 20–40 μg/kg/day (500–1000 times lower than conventional human therapeutic doses) prevented neuronal cell loss and striatal atrophy while improving motor function in the YAC128 mouse [157]. Lithium (lifelong 16 mg/kg/day s.c., corresponding to conventional human therapeutic oral doses) improved rotarod performance when treatment began post-symptomatically (but not pre-symptomatically) but there was no improvement in survival in R6/2 HD transgenic mice [165]. Combined treatment with lithium-plus-valproate was most effective, followed by lithium alone, but not valproate alone, each drug at human therapeutic levels (see Section 4.2), in improving motor skill learning and coordination tested by rotarod performance in the N171-82Q HD transgenic mouse [44]. Valproate (lifelong 100 mg/kg/day i.p. 5 days per week beginning at the age of 7 weeks, about 5 times human therapeutic oral doses) improved locomotor activity and markedly prolonged survival in the N171-82Q HD transgenic mouse [166]. In this same mouse model, combined treatment with lithium-plus-valproate at human therapeutic levels was most effective, followed by valproate alone, but not lithium alone, in markedly extending mouse survival time provided the treatment was started early at 7 weeks of age [44]. In this same study, in both N171-82Q and YAC128 HD transgenic mice, only combined lithium-plus-valproate treatment alleviated spontaneous locomotor deficits, and neither drug alone improved these deficits in either model [44].

The antidepressants imipramine 20 mg/kg/day, trazodone 20 mg/kg/day, and venlafaxine 10 or 20 mg/kg/day, doses 3–4 times human therapeutic doses, each improved impaired memory performance in both the Morris water maze and elevated plus maze escape retention test in the 3-nitropropionic acid rat HD model [152]. In two studies by the same group, these same doses of imipramine, trazodone, and venlafaxine each improved motor activity in the same rat model [153]. Nortriptyline given lifeflong at 10 mg/kg/day i.p. (7 times the human therapeutic oral dose) beginning at an age of 5 weeks delayed disease onset and improved motor performance in R6/2 mice [167]. Fluoxetine 20 mg/kg/day i.p. (at least 100 times human oral therapeutic doses) for 10 weeks reversed volume loss in the hippocampal dentate gyrus and improved cognitive and depressive behavior in R6/1 HD transgenic mice, although it did not affect motor activity or body weight [159]. In the 3-nitropropionate rat, sertraline improved impaired memory performance in both the Morris water maze and elevated plus maze escape retention test [152]. In the same model, two studies showed that sertraline 10 mg/kg/day (about 5 times human therapeutic oral doses) over 14 days also improved body weight, motor activity, and coordination [151,153]. In the N171-82Q HD mouse model, sertraline at clinically relevant doses (see Section 3) again attenuated striatal atrophy and behavioral abnormalities and increased survival [48]. In a magnetic resonance imaging (MRI) study of the N171-82Q HD transgenic mouse model, sertraline treatment (10 mg/kg/day i.p. beginning at 6 weeks) resulted in slowing of atrophy especially in the striatum [168,169] and frontal cortex [169] whereas there was no significant effect of coenzyme Q(10) treatment in these mice [169]. Sertraline for 10 weeks at clinically relevant doses (see Section 3) reduced striatal atrophy and was associated with improved motor performance and survival in R6/2 mice [47]. Melatonin lifelong at 30 mg/kg/day i.p. (about 1000 times human therapeutic doses) beginning at 6 weeks of age delayed disease onset and mortality in R6/2 mice [154].

In summary, the most promising findings at doses near clinical therapeutic ranges include replicated striatal neuroprotection and motor improvements with tetrabenazine (YAC128 mice), rotarod motor improvements with lithium (N171-82Q and R6/2 mice), locomotor improvement with imipramine, trazodone, and venlafaxine (nitropropionate rat), and, with sertraline, locomotor and rotarod improvements (nitropropionate rat) as well as striatal neuroprotection and extended survival (N171-82Q mice), although the lithium finding still awaits within-model replication. Single studies at near-clinical doses awaiting within-model replication include striatal neuroprotection, improved weight, and extended survival with lithium (nitropropionate and quinolinate rats), extended survival with valproate (N171-82Q mice), and motor and behavioral improvements with sertraline (N171-82Q mice). Findings replicated within models should subsequently be replicated across other HD transgenic mouse models at conventional human therapeutic doses and then considered for exploration in human HD.

## 9. Neuroprotective Disease-Modifying Clinical Trials of Psychotropics in HD

Thus far, there have been no controlled clinical trials of psychotropics in patients with HD that have employed designs that observe for disease modification. There is a suggestive case series regarding lithium and a negative clinical trial of the second-line mood stabilizer lamotrigine. A case series of three patients with HD chorea diagnosed by expanded CAG repeats has been reported, treated with low-dose lithium 150 mg/day and carbamazepine 200 mg bid in cases 1 and 2 and low-dose lithium 300 mg/day, carbamazepine 800 mg/day, quetiapine 300 mg/day, and clonazepam 2 mg/day in case 3, with nonprogression of chorea (all 3 cases) and dementia (case 3) over a follow-up of at least 3 years [170], although multiple drugs, limited observation periods, and the lack of blinding, objective measures, and controls limit conclusiveness. Although a number of psychotropic clinical trials are currently underway [171], none employ neuroprotective designs and none involve methodology sufficient to observe for neuroprotective disease modification (e.g., delayed start, randomized withdrawal paradigms), consistent with and unchanged from the conclusion of Mestre *et al.* in their 2009 Cochrane review of therapeutics for HD disease progression [172].

The only clinical trial conducted using a psychotropic has involved lamotrigine, an anticonvulsant that inhibits glutamate release and that is used as a second-line mood stabilizer in treating bipolar disorder. Kremer *et al.* [173] conducted a 30-month randomized, double-blind, placebo-controlled, parallel group trial of lamotrigine 400 mg/day in 64 patients with HD motor signs present for less than 3 years. The primary outcome was the Total Functional Capacity (TFC) score, with secondary outcomes including the Quantified Neurological Exam (QNE) and neuropsychological assessments (Mini-Mental State Exam (MMSE), Wechsler Adult Intelligence Scale-Revised (WAIS-R), Wechsler Memory Scale (WMS), paired associates, paragraph recall, word fluency, Beck Depression Inventory, Klove Grooved Pegboard, and finger-tapping tests). Assessments were conducted at baseline and at 12, 24, and 30 months. Although the study did not employ a disease-modifying design *per se*, slopes of deterioration were compared, and subjects were also evaluated by repeated fluorodeoxyglucose positron emission tomography (PET) for regional cerebral metabolism. Of the 28 lamotrigine and 27 placebo completers of the trial, there were no significant differences between groups on primary or secondary outcomes, with TFC deteriorating −1.89 ± 2.46 on lamotrigine *vs.* −2.11 ± 1.99 on placebo. Symptomatic improvement was seen with lamotrigine (53.6% *vs.* 14.8%, *p* = 0.006, for items including clumsiness, speech, balance, energy, and mood) with a trend toward decreased chorea (*p* = 0.08). In contrast to the pegboard (*p* < 0.001) and finger tapping tests (*p* < 0.01), global cognitive assessments were insensitive to deterioration over time. Among the 26 PET completers, the basal ganglia (*F* = 25.06, *p* < 0.001), frontal cortex (*F* = 6.52, *p* < 0.02), temporal cortex (*F* = 5.50; *p* < 0.05), and thalamus (*F* = 6.13, *p* < 0.05) showed reductions in metabolic rate over time, with the rate of reduction increasing over time. Thus, although this study did not find a neuroprotective effect of lamotrigine in HD, a symptomatic benefit was found.

Although not of psychotropic agents, previous neuroprotective clinical trials in HD provide experience applicable to designing future psychotropic disease-modifying clinical trials. Previous trials indicate the need for screening criteria and prioritization of validated mechanistic targets. Some screening and prioritization criteria [13] are adapted here for HD and offered in Table 4, and promising drugs can be further screened by futility trials using endpoints from historical controls [174], which are less costly to conduct than formal clinical trials. Another consideration would be to select candidate drugs with a given mechanism of action by their effect size in order to reduce sample size requirements and increase the odds of success, although this approach might prematurely exclude drugs with multiple mechanisms of action, including some mechanisms not yet elucidated. Still, animal models do not fully recapitulate human HD, and positive findings in animal and other preclinical models by no means assure similar results in patients with HD, as reflected in the clinical trials experience to date. Issues complicating trials have included the relatively low prevalence of HD, limited dose ranges due to side-effect profiles, study attrition over prolonged periods, dropouts due to adverse events, drug compliance, and variable blood levels of drug. Study attrition rates might be reduced by dedicated study coordinators [173] and the exclusion of advanced disease [175,176], but mild HD carries with it the trade-offs of slower and more varied progression that are associated with smaller between-group differences [175] and larger standard deviations in outcome measures [173], reducing study power to detect clinical effects. Clinical outcome measure issues limiting power have included insensitivity, floor and ceiling effects, susceptibility to subjective impression and placebo response, and unexpectedly large standard deviations [173].

Primary and secondary outcomes employed most commonly in randomized, double-blind, placebo-controlled, parallel group clinical trials observing for disease modification have included the Unified Huntington’s Disease Rating Scale (UHDRS) Total Functional Capacity (TFC) score, reduced form UHDRS motor component Total Motor Score-4 (TMS-4), MMSE, WAIS, Stroop Test, and Beck Depression Inventory [172]. The most common primary outcomes have included the UHDRS, TFC, and TMS-4. The TFC is a well-studied outcome, is clinically valid with good inter-rater reliability, and is easy to administer but can vary substantially between studies [175] and can be affected by a number of variables within a given study that include subject nurture, family support, employment situation, cognition, mood, other psychopathology, and drugs such as antipsychotics [173,175]. The TFC did not correlate with CAG repeat length in the lamotrigine study [173] but did correlate with paternal inheritance and younger age in the baclofen trial [175] which had been conducted before the discovery of the HTT gene CAG repeats. Other less well-studied outcomes include the TMS-4, TFC-plus-UHDRS, QNE, and HD Activities of Daily Living (HD ADL). The TMS-4 deteriorated approximately 4 points per year while most other UHDRS scales were insensitive to change over the 12-month ethyl-eicosapentaenoate study period [177]. UHDRS-related motor measures (TMS-4, TFC-plus-UHDRS), particularly chorea, can vary by subject relaxation state and, in later onset HD, CAG repeat length [177], although repeat length was not correlated in the predominantly early stage sample of the riluzole trial [176]. Power calculations have generally assumed a 50% slowing in HD progression, a standard that may be too high for likely effects. Although power calculations are not strictly comparable, it has been calculated that the number of study subjects needed for trials would be, for the TFC, 250 (80% power, alpha 5%, detecting 40% progression rate slowing over 18 months [175]) to 350 (80%, 5%, 40%, 30 months [178]), 135 for TMS-4 (80%, 5%, 12 point change, 12 months [177]), 300 for TFC-plus-UHDRS motor scores (90%, 5%, 36 month, 1 point change [176]), 340 for the QNE (90%, 5%, 50% slowing, 12 months [179]), and 260 for HD ADL (90%, 5%, 50% slowing, 12 months [179]). Cognitive outcome measures have been insensitive, correlated poorly with HD progression, are subject to practice effects, and need further study [173,180]. Frontal neuropsychological batteries have been suggested as better candidates for longitudinal assessments [173] although deterioration in the Symbol Digit Modalities Test, Stroop, and FAS Verbal Fluency tests was not appreciable over 12 months in the creatine study [180].

The study of presymptomatic HD and the use of time-to-event variables, survival times, failure times, and biomarkers represent strategies to enhance the power of investigations to detect delayed onset and progression of HD. The trade off of subject retention and reduced standard deviation *vs.* reduced mean change due to slower progression in early HD might be overcome by use of survival or failure time as a primary outcome [175]. Presymptomatic HD provides an opportunity for assessing delayed onset while time-to-event variables can provide an index of delayed progression. Sensitive, simple, specific, and robust biomarkers of disease modification that track disease activity, progression of disease severity, response to therapeutics, and predict conversion from presymptomatic to symptomatic disease would help to truncate trial durations, diminish sample size, increase power, and allow earlier conclusions regarding treatments than what is currently possible. Although the coefficient of variation (standard deviation divided by the mean) was lower and decline was greater for the PET measures than for the clinical measures in the lamotrigine study [173], PET can be confounded by pharmacological effects and cannot yet be considered to be a valid and reliable replicated biomarker. Biomarkers are discussed below (see Section 10).

In summary, the only psychotropic clinical trial observing for disease modification has been a negative study of lamotrigine, but experience gained from this and other trials of non-psychotropic agents provides useful guidance for selecting stage of illness and outcome measures in the design of future investigations with greater power to detect disease modification.

## 10. Conclusions and Research Directions

The positive findings for psychotropics in HD models are summarized in Tables 5–9. Given the frequency with which psychotropics are employed in the care of patients with HD, it will be important to advance research as has been detailed in each section above, and, beyond the drugs cited above, to now study in HD models additional psychotropics with desirable neuroprotective and epigenetic profiles. We have detailed elsewhere the neuroprotective actions of psychotropics in other neurodegenerative diseases [9–15]. Many psychotropics also have HD epigenetic target (Table 2) properties, which have been demonstrated in non-HD models and are too extensive to list here. Although currently cryptic, it should become clear with further study which of the psychotropic effects are therapeutic in HD and which instead constitute epiphenomena.

The most frequently demonstrated neuroprotective mechanisms across psychotropic classes involve mitochondrial protection and prevention of apoptosis. As seen in Tables 5–9, the most common mechanistic findings for different drug classes involve: mitochondrial protectant and antiapoptotic effects for neuroleptic antipsychotics; pro-autophagic, epigenetic, antiapoptotic, and neurogenic effects for the mood stabilizer lithium; histone acetylation and pro-BDNF effects for the mood stabilizer valproate; mitochondrial protectant and antiapoptotic effects for heterocyclic antidepressants and melatonin; and mitochondrial protectant and neurogenic effects for selective serotonin reuptake inhibitor (SSRI) antidepressants (the quality of these data is discussed below). In general, the clinical therapeutic psychiatric effects of neuroleptics correlate with dopamine D2 receptor antagonism, heterocyclic and certain other antidepressants with norepinephrine and serotonin reuptake inhibition (*i.e.*, norepinephrine and serotonin transporter binding), SSRIs with serotonin reuptake inhibition, antihistaminic sedation with histamine H1 receptor antagonism, and melatonin with MT1 melatonin receptor and gamma-aminobutyric acid receptor binding. Many mechanisms, yet to be resolved, have been proposed for the mood stabilizing effects of lithium and certain anticonvulsants. It is important to remember, however, that drugs within a psychopharmacological class can have markedly different properties beyond their unifying pharmacodynamic mechanisms, these drugs have been studied in an inconsistent and non-systematic fashion, and only certain drugs in certain classes have been studied in certain ways. Thus it is not possible to conclude, especially at this early point in their investigation, that psychotropic HD neuroprotective properties can be predicted by their psychopharmacodynamic mechanisms.

Since drugs can have multiple simultaneous beneficial and deleterious actions, some can have simultaneous contradictory effects on a single action, animal model and human responses can differ, and drug effects can vary by cell type, pathology, disease stage, neuronal location, neuronal maturity, drug dose, and drug treatment duration, clinical conclusions cannot be drawn from the preclinical data until human study is undertaken. Examples of variable outcomes include lithium’s opposite effects on AP-1 (Section 4.1), and on p53 (Section 4.4), under normal conditions *vs.* excitotoxic exposure. Even in the same model, a single drug may produce contradictory effects through different mechanisms, such as lithium’s ability to simultaneously upregulate and downregulate autophagy (Section 3). It is also not clear how well cell and tissue culture observations translate to animal models and human patients. HD animal models may also differ from human HD, and animal models do not fully recapitulate all attributes of human HD. For example, histone H3 is hypophosphorylated in the R6/2 HD transgenic mouse [66] although it is hyperphosphorylated in human HD [105]. Furthermore, negative results in animal models do not necessarily preclude the potential for positive results in human HD, for example sertraline’s lack of effect on huntingtin aggregates (see Section 3). These caveats notwithstanding, once current findings are replicated and found to be promising across multiple HD transgenic models, psychotropics with multiple neuroprotective mechanisms should be considered for trials in human patients with HD. In the current absence of psychotropic neuroprotective clinical trials, these preclinical findings, summarized in Tables 5–9, begin to define clear avenues for eventual translational clinical research in HD patients.

Disappointingly, the literature of psychotropic neuroprotection in HD is largely a series of promising but unreplicated findings. To review, there are as yet no data in HD models to indicate that individual psychotropics affect *HTT* or modifying gene transcription, CBP binding, miRNA, eRNA, REST, or HDAC although data from non-HD models suggest that these phenomena should be investigated in HD models at appropriate psychotropic doses and treatment durations. Although high dose sertraline failed to reduce huntingtin aggregation in either the R6/2 or N171-82Q transgenic mouse, unreplicated HD cell culture (high dose) and animal model (extreme dose) studies indicate that haloperidol reduced aggregation, while high dose lithium reduced aggregation in two HD cell culture models and stimulated autophagy in four, but replication is still needed at human therapeutic concentrations. It is unknown whether other SSRI antidepressants would behave similarly to sertraline. Lithium neuroprotectively downregulated AP-1 DNA binding at human therapeutic concentrations in an unreplicated study of neuronal culture exposed to glutamate. Lithium and valproate have each neuroprotectively increased histone H3K9 and H3K14 acetylation at human therapeutic concentrations in two different transgenic mouse models, awaiting within-model replications, while lamotrigine increased H3 and H4 acetylation in an unreplicated glutamate cell culture model. Downregulation of p53 at therapeutic lithium concentrations in cultured neurons exposed to glutamate suggests that treating HD transcriptional dysregulation by p53 upregulation may be inviable for lithium, however replication across HD models is needed. A single study demonstrated miR-222 upregulation with a combination of lithium and valproate at near-therapeutic concentrations in a glutamate-exposed neuronal culture, but each drug awaits individual investigation. Similarly, there primarily exist only single studies of various indices of mitochondrial protection and apoptosis for several antipsychotics, lithium (apoptosis only), antidepressants, and hypnotics at concentrations and doses within 1 order of magnitude of clinical therapeutic levels across HD striatal culture and animal models, each awaiting within-model replication. Replicated mitochondrial respiratory enzyme improvement seen with high-dose imipramine, trazodone, sertraline, and venlafaxine should be demonstrated beyond the nitropropionate rat model and at conventional therapeutic doses. BDNF upregulation has been evident in several transgenic mouse models for lithium, valproate, and sertraline at therapeutically-relevant doses, but still require replication within each model. More optimistically, the animal model literature has shown within-model replicated striatal neuroprotection and clinical disease modification at near-therapeutic doses for tetrabenazine (YAC128 HD transgenic mice), the antidepressants imipramine, trazodone, and venlafaxine (nitropropionate rat), and sertraline (N171-82Q and R6/2 HD transgenic mice, nitropropionate rat), and these findings should now be followed up on by extension in other transgenic mice at human therapeutic doses.

In the absence of disease modifying drugs in HD, and given the frequency at which psychotropics are prescribed in HD, it is worth finding out whether any of the above psychotropic therapeutic strategies will bear fruit. Obviously, more work is needed to replicate these positive findings at therapeutic doses before definite conclusions can be formulated regarding neuroprotective actions of antipsychotics (haloperidol, trifluoperazine, loxapine), mood stabilizers (lithium, valproate), antidepressants (imipramine, desipramine, nortiptyline, maprotiline, trazodone, sertraline, venlafaxine), and hypnotics (cyproheptadine, melatonin) in regard to huntingtin aggregation and autophagy, AP-1 binding, histone acetylation, p53 downregulation, miR-222, mitochondrial protection, apoptosis, and BDNF. Even upon replication, it still remains to be demonstrated that these effects actually mediate neuroprotection and are not just epiphenomena. On a more positive note, the more encouraging findings of striatal neuroprotection and disease modification seen in transgenic mice with tetrabenazine and antidepressants should be followed up and extended in additional transgenic models, hopefully with eventual exploration in human HD. The only psychotropic clinical trial thus far has involved lamotrigine, showing no evidence of clinical disease modification although PET findings were suggestive.

Although the frequency of psychotropic treatment in these patients suggests that retrospective analyses of large institutional databases might represent a practical approach, such studies will require careful subject selection and strenuous matching criteria to avoid negative results [181]. A particular obstacle to retrospective studies will be the confound of multiple drugs in the regimen [181]. The ideal investigations will be large prospective, multi-center, double-blinded, placebo-controlled studies with delayed-start or randomized-withdrawal designs to observe for disease-modifying neuroprotection, difficult to undertake in the current economic environment. Ultimately, once disease modification is demonstrated for several agents in patients with HD, it may even be possible to craft regimens that simultaneously provide symptomatic relief while synergizing the neuroprotective properties of each component drug. For example, chorea, psychosis, bipolar disorder, major depression, anxiety, irritability, and aggression are unusually common in HD [5–8]. A triple drug regimen clinical trial of trifluoperazine (to prophylax against chorea, psychosis, bipolar disorder, and aggression), lithium (bipolar disorder, depression, irritability, and aggression), and sertraline (depression, anxiety, irritability, and aggression) might be administered to presymptomatic patients with HD and compared to treatment-as-usual presymptomatic controls to observe for clinical and disease-modifying outcomes using time-to-event measures. Alternatively, tetrabenazine, lithium, and sertraline might be tried, possibly substituting valproate for, or adding valproate to, lithium. Although these regimens would appear to cover and synergize multiple neuroprotective mechanisms in HD, it will be important to first demonstrate efficacy and the absence of unforeseen pro-degenerative effects in HD animal models. This process may be facilitated by biomarkers of neuroprotective actions.

Biomarkers that correlate with disease modification and might reduce clinical trial duration and cost in HD include neuropsychological, biochemical, electroencephalographic (EEG), and neuroimaging measures. To date, most of the non-imaging markers suffer from a lack of reproducibility or absence of correlation with longitudinal illness course [182]. In regard to clinical and neuropsychological tests, longitudinal studies indicate non-linear motor and cognitive deterioration in presymptomatic individuals [183], lesser sensitivity of cognitive and motor markers than structural imaging to disease progression [184], and that the only significant longitudinally-correlated outcomes at one year (the indirect circle-tracing task and the chorea position index) seem to reflect stage-dependent phenotypes rather than pathobiological progression [184]. Biochemical markers await replication and consistent correlation with longitudinal illness course [182]. Peripheral blood BDNF is insensitive to HD progression [160]. The histone H2AFY is a candidate peripheral blood biomarker that has been cross-sectionally validated and replicated in patients over the HD course, longitudinally tracks the disease in R6/2 HD transgenic mice, and showed disease-modifying trajectories in a double-blind, placebo controlled, delayed start clinical trial of the HDACI sodium phenylbutyrate, but it remains to be longitudinally correlated with clinical and imaging markers of disease progression in HD prospective clinical trials [185]. EEG parameters display individual variability and require replication and longitudinal correlation [182]. Similar concerns apply to neuroimaging techniques assessing myelination, metabolism, and functional physiology in HD [186]. Although the coefficient of variation was lower and the decline was greater for the PET measures than for clinical measures in the lamotrigine study [173], PET can be confounded by pharmacological effects and cannot yet be considered to be a valid and reliable replicated biomarker in HD. Thus, most biomarkers are in various stages of development but are not yet ready to supplant clinical measures in following HD.

At the present time, structural MRI imaging is the best-established, most sensitive approach for following longitudinal HD progression. Across studies, basal ganglia structure volumes each correlate with multiple cognitive functions and measures [186] and are the most sensitive to disease progression [187,188], with caudate atrophy progressing more quickly (2.4%–4.1% per year) [189] and better predicting progression than other structures [190] in both presymptomatic and early HD. White matter degeneration also progresses over the course of HD [184,189,191] and is correlated with motor, cognitive, and functional measures [186]. Other structures and whole brain analyses are less well characterized [186]. Again, while a 50% slowing in progression is likely overly optimistic, in presymptomatic subjects, sample sizes for clinical trial arms to detect a 50% reduction in atrophy rate over 1 year have been calculated as 48 [187] and 103 [192] for the caudate, 58 putamen [192], 35 pallidum [192], and 61 white matter [189]. In early stage HD, the number needed to detect just a 40% slowing of caudate atrophy has been calculated as 89 subjects per arm [184], and 50% slowing of white matter atrophy has been calculated as 20 [193]. Thus, the best-established biomarker of HD progression is caudate atrophy, followed by white matter degeneration, with pallidal atrophy representing another attractive candidate once it is replicated. Caudate, and to a lesser extent, putamenal atrophy on MRI, correlate with CAG repeat size [189,191,194,195], and this variable should be considered in studies employing caudate volumetry.

The results reviewed here are subject to experimental and model limitations, a paucity of data, needed replication, the usual literature search caveats, and unproven translation to modifying human HD disease course. The literature reviewed was quite extensive, and the sheer volume along with literature database indexing inconsistencies and limited information in article abstracts will unavoidably lead to some literature being missed in this review. Obviously, further research is needed before undertaking prospective neuroprotective clinical trials of psychotropics in patients with HD. Thereupon, newer drugs with relevant neuroprotective mechanisms of action [13] might provide funded opportunities for eventual clinical trials. The wealth of potential neuroprotective properties of psychotropics, together with their frequency of use in patients with HD, makes further investigation a compelling priority.

## Figures and Tables

**Table 1:** Psychotropic classes and drugs investigated.

Psychotropic classes		Drugs investigated
**Antipsychotics**	**Neuroleptics**	Chlorpromazine Haloperidol Fluphenazine Thioridazine Thiothixene Trifluoperazine
	**Atypical antipsychotics**	Aripiprazole Asenapine Clozapine Iloperidone Lurasidone Olanzapine Paliperidone Quetiapine Risperidone Ziprasidone
	**Selective aerotonin 2A inverse agonists**	Pimavanserin
	**Vesicular monoamine transporter inhibitors**	Tetrabenazine
**Mood stabilizers**	**Anticonvulsants**	Carbamazepine Oxcarbazepine Lamotrigine Valproic Acid
	**Lithium**	Lithium
	**Other (pseudobulbar affect)**	Dextromethorphan/quinidine
**Antidepressants**	**Tricyclics**	Amitriptyline Clomipramine Desipramine Doxepin Imipramine Nortriptyline Protriptyline Trimipramine
	**Tetracyclics**	Maprotiline
	**Selective serotonin reuptake inhibitors**	Citalopram, Escitalopram Fluoxetine Fluvoxamine Paroxetine Sertraline
	**Serotonin and norepinephrine reuptake inhibitors**	Duloxetine Venlafaxine, Desvenlafaxine
	**Other**	Bupropion Mirtazapine Trazodone
**Anxiolytics**	**Antihistamines**	Cyproheptadine Diphenhydramine Hydroxyzine
	**Benzodiazepines**	Alprazolam Chlorazepate Chlordiazepoxide Clonazepam Diazepam Flurazepam Lorazepam Oxazepam Temazepam
	**Serotonin 1A receptor agonists**	Buspirone
**Hypnotics**	**Benzodiazepines**	See anxiolytics above
	**Non-Benzodiazepines**	Zaleplon Zopiclone, Eszopiclone Zolpidem
	**Antihistamines**	See anxiolytics above
	**Melatoninergics**	Melatonin Ramelteon
**Wake promoting agents**	**Non-sympathomimetic**	Modafinil, Armodafinil
**Anti-apathy agents**	**Dopaminergic agonists**	Amantadine Pramipexole Ropinirole

**Table 2:** Epigenetic targets in Huntington’s Disease.

Process		Epigenetic targets
**DNA methylation**	**Upregulate**	DNA methylation, especially in low CpG content regions [50] Transcriptional regulator SOX2 in low CpG content regions [50] 5-hydroxymethylcytosine levels in the *ADORA2A adenosine A2A receptor* gene 5′UTR region [51]
	**Downregulate**	Transcriptional regulator FRA-2 in low CpG content regions [50] Transcriptional regulator JUND in low CpG content regions [50] Transcriptional regulator AP-1 in low CpG content regions [50] 5-methylcytosine levels in the *ADORA2A adenosine A2A receptor* gene 5′UTR region [51] Alpha thalassemia/mental retardation X linked (ATRX) [52] Cdx-2 (especially activation, or its occupancy at the *ATRX* promoter) [52]
**Histone modification**	**Upregulate**	Chromatin unpacking into a de-repressed unpacked configuration [53] Ubiquitylation of mono-ubiquitylated histone H2A [54] Mono-ubiquitylation of histone H2B [54] Acetylation of histone H2A [55] Acetylation of histone H2B [55] Histone H3 [56] Acetylation of histone H3, especially at lysine 9 [55,57–62] Acetylation of histone H3, especially at lysine 14 [57–62] Acetylation of histone H4 [55,57–62] Acetylation of alpha-tubulin [63] Nuclear histone acetyltransferase (HAT or KAT) [53] CREB-binding protein CBP (KAT3A) [57,58,64] Phosphorylation of CBP [57,58] CREB-regulated transcription coactivator 1 TORC1 [65] p300/CBP-associated factor (P/CAF, or KAT2B) [57,64] Phosphorylation of histone H3 [56,66] Mitogen- and stress-activated kinase-1 MSK1 [66,67] Phosphorylation of MSK1 [56,66] Peroxisome proliferator activator receptor gamma (PPARgamma) [68] PPARgamma coactivator-1alpha (PGC1alpha) [69–71] Phosphorylation of PGC1alpha promoter [67] AMP-activated protein kinase AMPK [72] TAF4 [72] TAF4/CREB complex [72] SIRT1 [65,73,74] SIRT3 [75] Transcription factor p53 [58] in nucleus (not cytoplasm)
	**Downregulate**	Mono-ubiquitylation of histone H2A [54] Histone methyltransferases Methylation of histone H3, especially at lysine 4 (K4) [54,76] Methylation of histone H3 lysine 9 (K9), especially *tri*methylation at lysine 9 [54,76–78] ERG-associated protein with SET domain (ESET/SETDB1, also known as KMT1E) [77] DNA binding of specificity proteins Sp1 and Sp3 [77] Huntingtin-interacting protein SETD2/HYPB, responsible for H3K36 trimethylation [79] Histone deacetylases (HDAC) [57,59,76] HDAC1 [80] HDAC2 [81,82] HDAC3 [83] HDAC4 [56,81,82] HDAC5 [55,84] HDAC6 [63] HDAC11 [81,82] SIRT2 [85] Transglutaminase2 [86] Transcription repressor element-1 (RE1) silencing transcription factor (REST, also known as neuron-restrictive silencing factor (NRSF)) [87]
**Posttranscriptional RNA editing**	**Upregulate**	Posttranscriptional RNA editing of glutamate receptor GluR-2 [88,89] Posttranscriptional RNA editing of glutamate receptor GluR-6 [88,89]
**MicroRNA**	**Upregulate in presymptomatic and early HD**	miR-135b [90] and miR-212 [90,91]
	**Downregulate in presymptomatic and early HD**	miR-29a [90–92], miR-34b [93], miR-200a [94], and miR-200c [94]
	**Upregulate**	miR-9 [90,91,95], miR-9* (miR-9A) [90,91,95], miR-22 [96,97], miR-29b [90], miR-29c [96], miR-100 [95], miR-124 [98], miR-124a [90], miR-125b [95,99], miR-128 [96], miR-132 [96,100], miR-135a [95], miR-135b [95], miR-137 [101], miR-138 [95,96], miR-139 [90,91], miR-146a [95,99], miR-150 [95,99], miR-181c [95], miR-190 [95], miR-196a [102], miR-218 [91,95,96], miR-221 [95], miR-222 [95,96], miR-330 [91], miR-338-3p [95], miR-344 [96], miR-485 [103], miR-674 [96], ban [104], let-7a [91], let-7c [91], let-7d [91], and let-7e [91]
	**Downregulate**	miR-30a [91], miR30b [91], miR-30c [91], miR-30e [91], miR-34b [93], miR-127-3p [95], late HD cortical miR-132 [90,91], miR-145 [95], miR-148a [95], miR-199-3p [95], miR-199-5p [95], miR-200a [95], miR-205 [95], miR-214 [95], and miR-335-5p [95]

Epigenetic targets based on data obtained in Huntington’s disease models, including some targets awaiting replication and establishment. Some of the targets may represent epiphenomena unrelated to HD pathogenesis.

**Table 3:** Psychotropic effects on Histone Deacetylases (HDACs).

EFFECT	HDAC1	HDAC2	HDAC3	HDAC4	HDAC5	HDAC6	HDAC11
**Downregulate**							
Carbamazepine	[135]						
Valproate	[136–138]	[137,139]		[140]			
Amitriptyline			[141]			[141]	
**Upregulate**							
Clozapine		[142]	[142]				
Olanzapine		[142]	[142]		[142]		
Lurasidone	[143]	[143]			[142,143]		
Carbamazepine		[142]	[142]		[142]		
Valproate					[143]		[144]
Lamotrigine		[142]	[142]		[142]		
Clomipramine		[142]	[142]				
Fluoxetine		[145]					
Escitalopram		[142]	[142]		[142]		
Duloxetine		[142]	[142]		[142]		
Mirtazapine		[142]	[142]		[142]		

Psychotropic downregulating and upregulating effects on histone deacetylases 1–6 and 11 or their function, demonstrated in non-HD models and awaiting confirmation in Huntington’s disease models.

**Table 4:** Criteria for determining candidate agents for clinical trials in HD.

Evidence type	Neuroprotective candidate attribute
**Preclinical criteria**	Experimental evidence of a neuroprotective action using an established neuroprotective model at physiological drug doses Independent replication of the neuroprotective action in the same model and at the same dose Replication of the neuroprotective action in at least one other model at a physiological dose Replication of the neuroprotective action in neural tissue, preferably mature neurons or glia, at a physiological dose Independent replication of the neuroprotective action in the specified neural tissue at the same site and in the same animal at the same dose (e.g., replication of a rat striatal neuron finding in a rat striatal neuron model, rather than in rat cerebellar granule cells) Evidence in an accepted animal model for HD (e.g., transgenic mice, quinolinate rat model, *etc.*, their limitations notwithstanding) Evidence of *multiple* neuroprotective actions that have been demonstrated as above A greater overall neuroprotective positive “valence” (the number of neuroprotective actions minus the number of neurodegenerative actions demonstrated for the agent of interest [e.g., an agent with 3 distinct neuroprotective actions and 1 neurodegenerative action might be assigned a positive valence of 3 − 1 = 2 (recognizing at the present time that different actions may someday be demonstrated to have differentially weighted correlation coefficients with neurodegenerative progression)])
**Clinical criteria**	Clinical evidence suggestive of delayed progression (e.g., lack of progression in chorea and dementia over 5 years, lack of deterioration in UHDRS TFC score after 5 years in a patient rigorously diagnosed for HD, absence of change in MRI caudate volume over 5 years in a patient rigorously diagnosed for HD, *etc.*) Evidence of a more benign disease course than expected for patients in a case series or clinical trial (particularly if symptomatic effects of the drug can be controlled for)

These are putative criteria for assessing the probability that an agent will demonstrate a translational neuroprotective effect in an HD clinical trial. Since the predictive utility of these factors remains to be demonstrated, an equal weighting may be assigned to each criterion. It is likely that the more criteria an agent meets, the greater the likelihood that it will yield significant results in a clinical neuroprotective trial in HD. Abbreviations: HD Huntington’s Disease; μM micromolar; mg milligram; ml milliliter; mM millimolar; MMSE Mini-Mental State Examination; MRI magnetic resonance imaging; TFC Total Functional Capacity; UHDRS Unified Huntington’s Disease Rating Scale (adapted from Lauterbach *et al.* [13]).

**Table 5:** Antipsychotic and tetrabenazine neuroprotective effects in Huntington’s disease models.

Neuroprotective action	D2 antagonist antipsychotics	Trifluoperazine	Haloperidol	Loxapine	Tetrabenazine
Huntingtin clearance	+		+		
AP-1 DNA binding					
Histone acetylation					
p53					
MiR-222					
Mitochondria	+	+	+		
Apoptosis		+		+	
Neurogenesis					
BDNF					
Striatal preservation			+		+
Cognitive integrity					
Motor integrity					+
Delay of disease onset					
Enhanced survival					

**Table 6:** Mood stabilizer neuroprotective effects in Huntington’s disease models.

Neuroprotective action	Lithium	Valproate	Lamotrigine
Huntingtin clearance by autophagy	+		
AP-1 DNA binding	+		
Histone acetylation	+	+	+
p53	+		
MiR-222	+? (when combined with valproate)	+? (when combined with lithium)	
Mitochondria			
Apoptosis	+		
Neurogenesis	+		
BDNF	+	+	
Striatal preservation	+		
Cognitive integrity			
Motor integrity	+	+	
Delay of disease onset			
Enhanced survival	+	+	

**Table 7:** Tricyclic and tetracyclic antidepressant neuroprotective effects in Huntington’s disease models.

Neuroprotective action	Imipramine	Desipramine	Nortriptyline	Maprotiline
Huntingtin clearance				
AP-1 DNA binding				
Histone acetylation				
p53				
MiR-222				
Mitochondria	+	+	+	+
Apoptosis		+	+	+
Neurogenesis				
BDNF				
Striatal preservation				
Cognitive integrity	+			
Motor integrity	+		+	
Delay of disease onset			+	
Enhanced survival				

**Table 8:** Other antidepressant neuroprotective effects in Huntington’s disease models.

Neuroprotective action	Trazodone	Fluoxetine	Sertraline	Venlafaxine
Huntingtin clearance				
AP-1 DNA binding				
Histone acetylation				
p53				
MiR-222				
Mitochondria	+		+	+
Apoptosis				
Neurogenesis		+	+	
BDNF			+	
Striatal preservation			+	
Cognitive integrity	+	+	+	+
Motor integrity	+		+	+
Delay of disease onset				
Enhanced survival			+	

**Table 9:** Anxiolytic and hypnotic neuroprotective effects in Huntington’s disease models.

Neuroprotective action	Cyproheptadine	Melatonin
Huntingtin clearance		
AP-1 DNA binding		
Histone acetylation		
p53		
MiR-222		
Mitochondria		+
Apoptosis	+	+
Neurogenesis		
BDNF		
Striatal preservation		
Cognitive integrity		
Motor integrity		
Delay of disease onset		+
Enhanced survival		+
